# Successful Valve-in-Valve-in-Valve Procedure in a Patient With Severe Aortic Prosthesis Dysfunction: A Case Report

**DOI:** 10.7759/cureus.106304

**Published:** 2026-04-01

**Authors:** Ramiz Emini, Dalal S Alshammari, Fayez A Alshammari

**Affiliations:** 1 Cardiac Surgery, Hail Cardiac Center, Hail, SAU; 2 Cardiology, Hail Cardiac Center, Hail, SAU

**Keywords:** aortic regurgitation, patient-prosthesis-mismatch, self-expanding vs. balloon-expanding valve, transcatheter aortic valve replacement, valve-in-valve-in-valve, valvular/paravalvular leak

## Abstract

Transcatheter aortic valve implantation (TAVI) has become an established treatment for patients with severe aortic stenosis, particularly those with elevated surgical risk. As indications for TAVI expand and patient survival improves, an increasing number of individuals may outlive their initial transcatheter heart valve (THV), creating new challenges in long-term valve management. Repeat procedures such as valve-in-valve implantation are therefore becoming more common. However, repeated transcatheter valve implantation increases the risk of complications, including patient-prosthesis mismatch (PPM), impaired coronary access, and residual or recurrent aortic regurgitation (AR). We report a rare case of early failure of two balloon-expandable transcatheter valves resulting in severe transvalvular regurgitation and recurrent heart failure. The patient was successfully treated with implantation of a third prosthesis using a self-expanding supra-annular valve in a valve-in-valve-in-valve (ViViV) configuration. This strategy corrected the regurgitation while preserving an adequate effective orifice area. This case highlights the importance of individualized valve selection and demonstrates that switching valve platforms from balloon-expandable to self-expanding devices may provide an effective solution in complex redo TAVI procedures.

## Introduction

Transcatheter aortic valve implantation (TAVI) has become an established treatment for patients with severe aortic stenosis, particularly those who are considered high risk for conventional surgical aortic valve replacement (SAVR) [1]. Compared with surgery, TAVI offers shorter recovery time and improved early outcomes in selected patients [2,3]. As the indications for TAVI continue to expand and patient survival improves, an increasing number of individuals are expected to outlive their initially implanted transcatheter heart valve (THV) [2,3].

The concept of lifetime management of patients undergoing TAVI has therefore become increasingly important. When a previously implanted prosthesis degenerates or becomes dysfunctional, repeat interventions, such as valve-in-valve procedures, may be required. Although repeat transcatheter valve implantation is technically feasible, it introduces additional challenges, including the risk of patient-prosthesis mismatch (PPM), impaired coronary access, elevated transvalvular gradients, and the possibility of residual or recurrent aortic regurgitation (AR) [4]. Optimal procedural planning is therefore essential during both the initial and repeat interventions. Factors such as prosthesis design, leaflet position, and valve expansion mechanism may significantly influence hemodynamic performance after implantation. Balloon-expandable valves typically function within the annular plane, whereas self-expanding valves are deployed in a supra-annular position [5,6].

In this report, we present a rare and challenging case of early failure of two balloon-expandable transcatheter valves resulting in severe transvalvular regurgitation and recurrent heart failure. The condition was successfully treated using a valve-in-valve-in-valve (ViViV) strategy with implantation of a self-expanding supra-annular prosthesis. This case highlights the importance of individualized procedural strategies and demonstrates how changing valve platforms may restore adequate valve function in complex redo TAVI procedures. This article was previously presented as a meeting abstract at the 2nd Hail Cardiology Conference on May 3, 2024.

## Case presentation

A 74-year-old woman was managed at the Hail Cardiac Center, Hail, Saudi Arabia, for severe symptomatic aortic stenosis. Her predicted surgical risk was high, with a Society of Thoracic Surgery (STS) score of 12.43% and a logistic EuroSCORE II of 8.99%, both indicating a significantly elevated risk of mortality with conventional surgical aortic valve replacement (SAVR). Therefore, the multidisciplinary heart team recommended transcatheter aortic valve implantation (TAVI). In December 2023, the patient underwent TAVI using a 23-mm balloon-expandable transcatheter heart valve (THV).

Due to initial prosthesis malposition and significant paravalvular leakage, a second valve was immediately implanted in a valve-in-valve configuration. At the end of the procedure, only mild residual aortic regurgitation (AR) was observed and was considered acceptable by the heart team. However, three months later, the patient developed recurrent cardiac decompensation characterized by dyspnea, peripheral edema, mild chest pain, and pleural effusion. She was admitted to the New York Heart Association (NYHA) functional class IV.

Initial management focused on optimization of guideline-directed medical therapy (GDMT) for congestive heart failure. Baseline laboratory investigations are summarized in Table 1.

**Table 1:** Baseline laboratory investigations.

Laboratory test	Patient value	Reference range	Unit	Interpretation
Hemoglobin	12.4	12–16	g/dL	Within normal range
White blood cells (WBC)	7.2	4.0–10.0	×10⁹/L	Normal
Platelet count	235	150–400	×10⁹/L	Normal
Serum creatinine	0.9	0.6–1.2	mg/dL	Normal renal function
Blood urea nitrogen (BUN)	18	7–20	mg/dL	Normal
Sodium	138	135–145	mmol/L	Normal
Potassium	4.2	3.5–5.0	mmol/L	Normal
B-type natriuretic peptide (BNP)	850	<100	pg/mL	Elevated, consistent with heart failure
High-sensitivity troponin	8	<14	ng/L	Within normal range
C-reactive protein (CRP)	4	<10	mg/L	No significant inflammation
International normalized ratio (INR)	1.0	0.8–1.2	-	Normal coagulation

**Figure 1:**
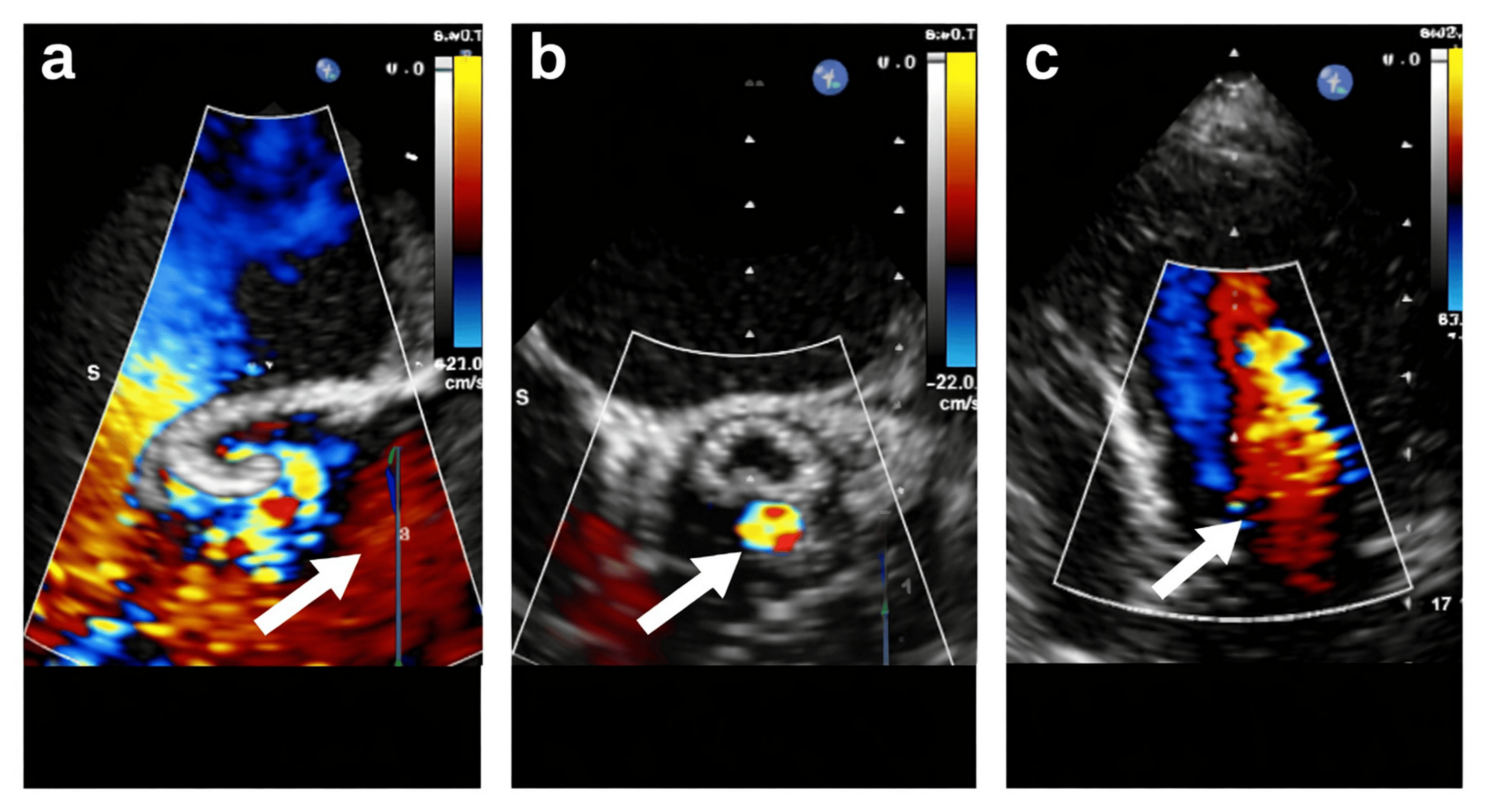
Echocardiographic evaluation of prosthetic valve dysfunction and post-procedural outcome. (a) Pre-procedural transesophageal echocardiography demonstrating severe transvalvular aortic regurgitation, with a prominent color Doppler jet directed into the left ventricle (arrow). (b) Transesophageal short-axis view demonstrating paravalvular leakage around the prosthetic valve visualized by color Doppler imaging (arrow). (c) Post-procedural transthoracic echocardiography demonstrating resolution of severe regurgitation after implantation of the third valve, with only mild residual paravalvular leakage (arrow).

Comprehensive evaluation using transthoracic echocardiography, transesophageal echocardiography, and cardiac magnetic resonance imaging revealed severe transvalvular AR and two small paravalvular leaks, resulting in a total regurgitant fraction of approximately 35%, as shown in Figure 1.

**Figure 2:**
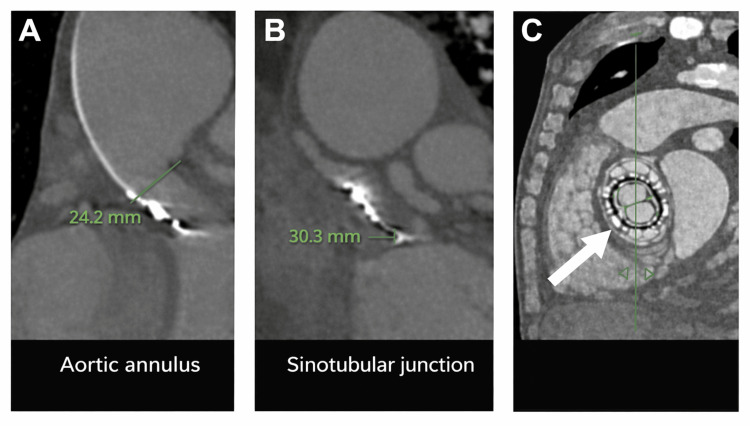
Multidetector computed tomography assessment of the aortic root. Multidetector computed tomography (MDCT) was performed using a 256-slice scanner (Philips Brilliance iCT), demonstrating measurements of the aortic root structures used for procedural planning prior to transcatheter valve implantation. (A) CT image demonstrating measurement of the aortic annulus diameter (24.2 mm) used for prosthetic valve sizing. (B) CT image demonstrating measurement of the sinotubular junction diameter (30.3 mm), an important anatomical landmark for valve positioning. (C) Multiplanar CT reconstruction demonstrating the spatial orientation and positioning of the prosthetic valve within the aortic root (arrow). These images represent original CT images obtained from the 74-year-old woman patient described in this case report, and written informed consent for publication was obtained.

A computed tomography (CT) scan using a 256-slice multislice scanner (Philips Brilliance iCT, Philips Healthcare, Best, The Netherlands) demonstrated extensive calcification of the ascending aorta consistent with a porcelain aorta, significantly increasing the risk of open surgery. CT findings are presented in Figure 2.

**Figure 3:**
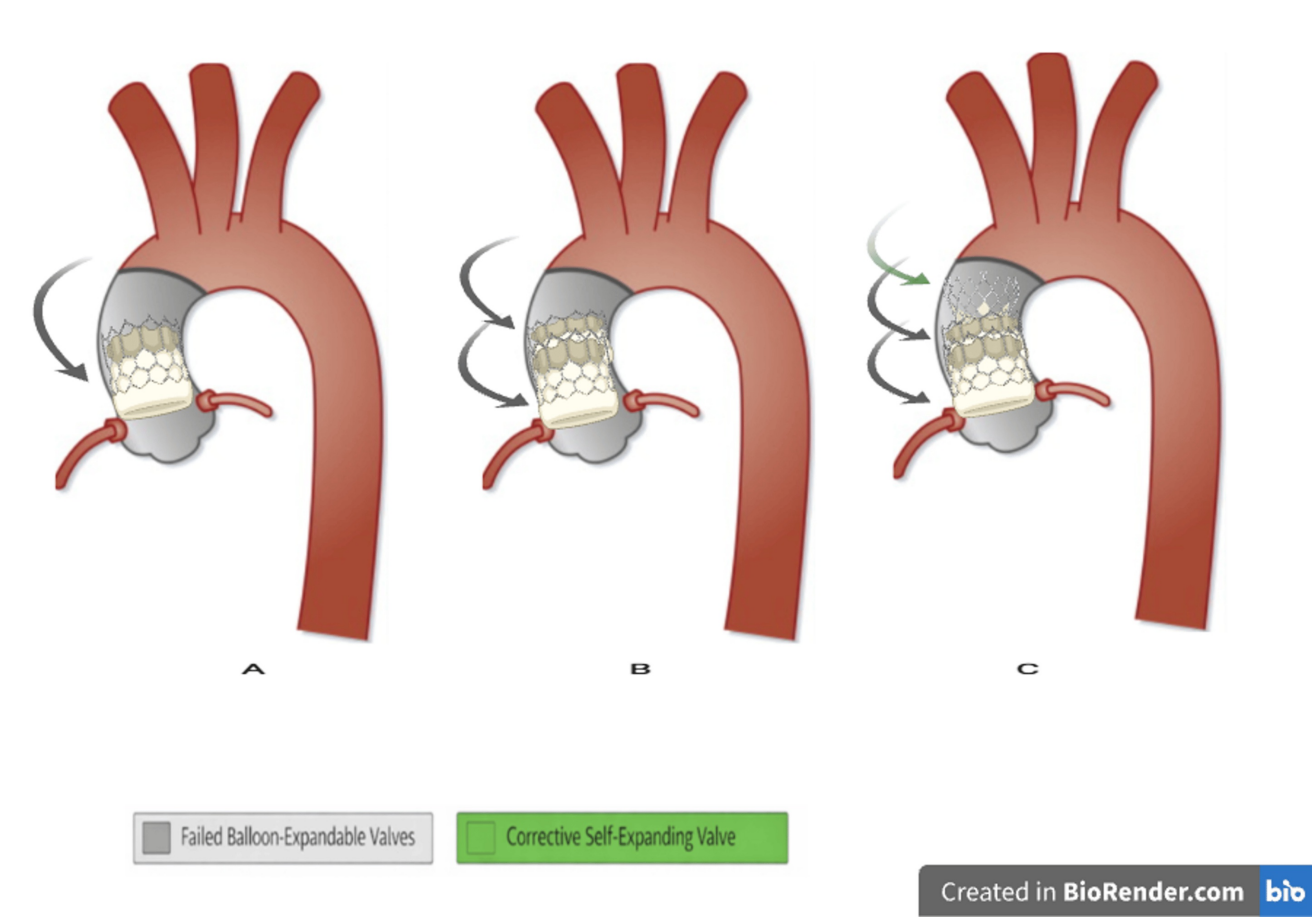
Schematic illustration of the valve-in-valve-in-valve (ViViV) configuration. (A) Implantation of the first balloon-expandable transcatheter aortic valve, positioned intra-annularly within the native aortic annulus. (B) Implantation of a second balloon-expandable valve as a valve-in-valve procedure, also positioned intra-annularly, was performed to treat prosthetic valve dysfunction with persistent aortic regurgitation. (C) Implantation of a self-expanding transcatheter valve in a supra-annular position, placed above the previously implanted valves, to restore valve competence and improve the effective orifice area. Gray prostheses represent failed balloon-expandable valves, whereas the green prosthesis indicates the corrective self-expanding valve. Figure was created by the authors using the BioRender platform (BioRender.com Inc., Toronto, ON, Canada).

Given the patient's comorbidities, surgery was considered prohibitive. Therefore, a third transcatheter valve implantation was planned. The procedure was performed percutaneously via the right femoral artery, and a self-expanding prosthesis was implanted, creating a valve-in-valve-in-valve (ViViV) configuration, illustrated in Figure 3.

**Figure 4:**
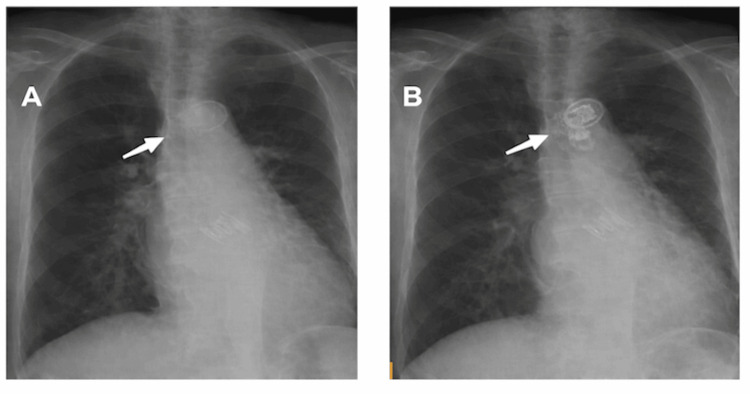
Chest radiographs demonstrating valve-in-valve-in-valve configuration. (A) Pre-procedural chest radiograph demonstrating the previously implanted balloon-expandable transcatheter aortic valve in the aortic position. (B) Post-procedural chest radiograph obtained after implantation of the third valve, demonstrating the valve-in-valve-in-valve (ViViV) configuration. Arrows indicate the location of the prosthetic valve complex within the aortic root.

Post-procedural chest radiography confirming correct valve positioning is shown in Figure 4.

The procedure was uneventful. The postoperative course was uncomplicated. At 18-month follow-up, the patient showed marked clinical improvement, with a left ventricular ejection fraction of 65% and functional status improved to NYHA class I. Dual antiplatelet therapy was prescribed for six months in addition to GDMT. The third valve implantation successfully restored valve competence and resulted in excellent clinical recovery.

## Discussion

Aortic regurgitation (AR) following transcatheter aortic valve implantation (TAVI) is a recognized complication that can negatively affect clinical outcomes [1]. In the present case, a 74-year-old woman with severe aortic stenosis underwent TAVI using two balloon-expandable prostheses but subsequently developed recurrent cardiac decompensation due to significant AR. This case highlights the complexity of managing patients who have undergone multiple valve implantations and emphasizes the importance of considering alternative strategies when conventional approaches fail.

Previous studies have demonstrated that TAVI prostheses with supra-annular leaflet positioning, such as the Acurate Neo and Evolut R/Pro systems, are associated with lower transvalvular gradients and larger indexed effective orifice area (iEOA) compared with intra-annular valve designs, thereby reducing the risk of patient-prosthesis mismatch (PPM) [4,5]. In contrast, balloon-expandable valves, which function at the annular level, are more frequently associated with PPM than self-expanding supra-annular valves [6]. Accordingly, the use of supra-annular valve designs has been recommended in patients at increased risk of PPM [6]. In our patient, implantation of a self-expanding prosthesis in a valve-in-valve-in-valve configuration within the supra-annular position resulted in restoration of valve competence and improved hemodynamics.

A clear and standardized definition of PPM, along with consistent recommendations regarding the use of predicted versus measured iEOA, is essential to improve comparability across studies. Establishing uniform criteria applicable to both TAVI and surgical aortic valve replacement (SAVR) would facilitate more accurate comparisons between treatment strategies [6].

Echocardiography remains the primary imaging modality for assessing prosthetic valve function following both TAVI and SAVR. PPM is typically defined based on the indexed effective orifice area (iEOA), calculated as the ratio of effective orifice area (EOA) to body surface area (BSA) [6,7].

This case also underscores the importance of comprehensive pre-procedural evaluation, including detailed echocardiographic and computed tomography (CT) imaging, to optimize procedural planning and improve outcomes [4].

Prediction of the iEOA prior to intervention, using reference tables derived from echocardiographic data, represents an important strategy to reduce the risk of PPM. When implantation of an adequately sized prosthesis is not feasible, alternative strategies, such as annular enlargement or transcatheter approaches, may be considered. Current European Society of Cardiology (ESC)/European Association for Cardio-Thoracic Surgery (EACTS) guidelines recommend TAVI as a preferred option in patients with a small aortic root, and appropriate valve selection, sizing, and procedural techniques such as post-dilatation may help minimize PPM [8,9].

In this case, a comprehensive clinical and imaging evaluation identified significant prosthetic valve dysfunction, which was successfully managed through the strategic use of a self-expanding valve system.

Overall, the successful correction of AR using a supra-annular self-expanding prosthesis resulted in significant clinical improvement and aligns with emerging evidence supporting individualized valve selection strategies in complex TAVI cases [4,9]. Further studies are required to better define optimal management strategies for patients with challenging anatomical and procedural characteristics.

This case report has several limitations. First, it describes the clinical course of a single patient, which limits the generalizability of the findings. Second, although the patient demonstrated significant clinical improvement, the follow-up duration of 18 months remains relatively short for assessing the long-term durability of a valve-in-valve-in-valve configuration. Larger studies with longer follow-up periods are required to better evaluate the long-term outcomes of repeated transcatheter valve implantation strategies.

## Conclusions

Aortic regurgitation following the implantation of two balloon-expandable valves led to recurrent episodes of cardiac decompensation in this patient. This case report illustrates the feasibility of transitioning from balloon-expandable to self-expanding valve technologies as a corrective measure for valve-in-valve-in-valve in the context of severe transvalvular leakage without further decreasing the effective orifice area. The problem of achieving sufficient valve closure with the largest feasible EOA was effectively resolved by the subsequent implantation of a supra-annular self-expandable prosthesis. With an optimized preoperative diagnostic approach and a good risk-benefit analysis for subsequent procedures, this research emphasizes how to maximize valve performance by utilizing the various types of prostheses and their anchoring and sealing positions.
